# Hyperforin/HP-*β*-Cyclodextrin Enhances Mechanosensitive Ca^2+^ Signaling in HaCaT Keratinocytes and in Atopic Skin Ex Vivo Which Accelerates Wound Healing

**DOI:** 10.1155/2017/8701801

**Published:** 2017-01-22

**Authors:** Hiroya Takada, Jun Yonekawa, Masami Matsumoto, Kishio Furuya, Masahiro Sokabe

**Affiliations:** ^1^Department of Physiology, Nagoya University Graduate School of Medicine, 65 Tsurumai, Nagoya 466-8550, Japan; ^2^Pixy Central Research Institute, 3-7-1 Kamitsuchidananaka, Ayase, Kanagawa 252-1113, Japan; ^3^Mechanobiology Laboratory, Nagoya University Graduate School of Medicine, 65 Tsurumai, Nagoya 466-8550, Japan

## Abstract

Cutaneous wound healing is accelerated by mechanical stretching, and treatment with hyperforin, a major component of a traditional herbal medicine and a known TRPC6 activator, further enhances the acceleration. We recently revealed that this was due to the enhancement of ATP-Ca^2+^ signaling in keratinocytes by hyperforin treatment. However, the low aqueous solubility and easy photodegradation impede the topical application of hyperforin for therapeutic purposes. We designed a compound hydroxypropyl-*β*-cyclodextrin- (HP-*β*-CD-) tetracapped hyperforin, which had increased aqueous solubility and improved photoprotection. We assessed the physiological effects of hyperforin/HP-*β*-CD on wound healing in HaCaT keratinocytes using live imaging to observe the ATP release and the intracellular Ca^2+^ increase. In response to stretching (20%), ATP was released only from the foremost cells at the wound edge; it then diffused to the cells behind the wound edge and activated the P2Y receptors, which caused propagating Ca^2+^ waves via TRPC6. This process might facilitate wound closure, because the Ca^2+^ response and wound healing were inhibited in parallel by various inhibitors of ATP-Ca^2+^ signaling. We also applied hyperforin/HP-*β*-CD on an ex vivo skin model of atopic dermatitis and found that hyperforin/HP-*β*-CD treatment for 24 h improved the stretch-induced Ca^2+^ responses and oscillations which failed in atopic skin.

## 1. Introduction

Epidermal keratinocytes are located at the surface of the skin and are exposed to various environmental stimuli including mechanical and physical stimuli and are susceptible to these stimuli. During the wound healing process, these exogenous stimuli and the endogenous stimuli, such as the tension and traction forces generated between the migration of the foremost cells and the cells that are located behind them, may affect the rate of wound closure. Our earlier study demonstrated that mechanical stretching facilitated wound closure in bovine aortic endothelial cells [1]. We recently reported that wound healing in HaCaT keratinocytes was accelerated by stretching and treatment with hyperforin, which is a major component of a traditional herbal medicine and which is known to be a TRPC6 activator, further accelerated wound closure [2]. We revealed that the facilitation of wound closure by mechanical stretching and hyperforin occurs due to the release of ATP via mechanosensitive hemichannels at the wound edge and the P2Y receptor-mediated Ca^2+^influx* via* TRPC6 in the cells located behind the wound edge using real-time ATP luminescence imaging and Ca^2+^ fluorescence measurement [2]. The influx of Ca^2+^ through TRPC6 channels was also reported to be essential for wound healing in vivo in TRPC6 knockout mice [3].

Hyperforin is a major active constituent of St. John's wort (*Hypericum perforatum *L.) extract, which is widely used in traditional herbal medicines, to promote wound healing [4–9]. The use of hyperforin-rich cream as a topical medication for atopic dermatitis was recently reported [10–14]. In spite of its potential therapeutic activities, the extreme sensitivity of hyperforin to photodegradation has impeded its topical application. The complexation of St. John's wort extract with *β*- and *γ*-cyclodextrin (CD) was reported to enhance the photoprotection and solubility of hyperforin in aqueous solutions [15–17]. In the present study, we aimed to develop a novel formation of encapsulated hyperforin with hydroxypropyl-*β*-cyclodextrin (HP-*β*-CD) to improve its aqueous solubility and photostability, because HP-*β*-CD has been shown to possess the highest solubility not only in water but also in ethanol among several of the CD compounds that are commonly used. We also assessed the effects of the compound on the wound healing and ATP-Ca^2+^ signaling in HaCaT keratinocytes.

Atopic dermatitis is a chronic inflammatory skin disease that develops due to various factors that are associated with epidermal barrier dysfunction [18]. It is known that the Ca^2+^ gradient in the epidermis is necessary for maintaining the barrier function; however, the Ca^2+^ dynamics of atopic skin remain to be elucidated. We herein measured the stretch-induced Ca^2+^ responses ex vivo in atopic skin using a confocal microscope. We found that the Ca^2+^ responses were impaired in the atopic epidermis and that the responses recovered after the application of hyperforin/HP-*β*-CD. The data suggested that hyperforin/HP-*β*-CD is a potent targeted therapeutic agent that can be used to promote epidermal wound healing and treat atopic dermatitis.

## 2. Material and Methods

### 2.1. Reagents

Hyperfolin/hydroxypropyl-*β*-cyclodextrin was prepared by the complexation of hyperforin (Cayman Chemical, Ann Arbor, MI) and hydroxypropyl-*β*-cyclodextrin (HP-*β*-CD; CycloChem, Tokyo, Japan) as described below. The other chemicals and reagents were as follows: carbenoxolone disodium salt (CBX), apyrase (from potato), GdCl_3_, U73122, and Cremophor EL (Sigma-Aldrich, St. Louis, MO); ionomycin (Calbiochem, San Diego, CA); suramin hexasodium (RBI, Natick, MA); GsMTx-4 (Peptide Institute, Osaka, Japan); diC8-PIP_2_ (Echelon Biosciences, Salt Lake City, UT); dispase (Godo Shusei, Tokyo, Japan); Fluo-8 AM (AAT Bioquest, Sunnyvale, CA); Cellmatrix type IA (Nitta Gelatin, Osaka, Japan); Lipofectamine (18324, Invitrogen, Carlsbad, CA); DME/F12 (D9785; Sigma-Aldrich, St. Louis, MO); FBS (12483; Gibco, Carlsbad, CA).

### 2.2. The Preparation of Hydrophilic and Stable Hyperforin/HP-*β*-CD

Solutions of 4.66 × 10^−4^ M hyperforin (in 1 mL methanol) and 1.86 × 10^−3^ M HP-*β*-CD (in 1 mL ethanol) were mixed and then stirred for 30 min. The solvents were then removed in vacuo with a centrifugal evaporator (0.1 Mpa, 2800 rpm, 90 min, WKN-PV-1200, Wakenyaku, Kyoto, Japan) at ambient temperature. The obtained white solid was dissolved in Milli-Q water by ultrasonication for at least 10 min. The resulting aqueous solution of hyperforin/HP-*β*-CD was syringe-filtered with a 0.20 *μ*m pore size and kept in a freezer until use. All of the procedures were performed under light-shielded conditions.

Stoichiometry of the reaction between hyperforin and HP-*β*-CD was spectroscopically determined. The concentration of hyperforin in these studies was 4.66 × 10^−4^ M whereas the HP-*β*-CD concentration was used in the range of 0–8.0 equivalents. The UV spectra of hyperforin were recorded using a UV/VIS scanning spectrophotometer (Gene Spec III, Hitachi Naka Instruments, Hitachinaka, Japan). The changes in the absorbance of hyperforin following the addition of various concentrations of the HP-*β*-CD complexing agent were measured at *λ*_max⁡_ 281 ± 7 nm.

### 2.3. The Analysis of Irradiated Hyperforin Solution by HPLC

An irradiation test was performed using a 6-watt LED light bulb (total luminous flux 480 lm, color temperature: 6700 K, Panasonic, Osaka, Japan) that was placed 14 cm above the samples. Irradiation was conducted in a dark room under temperature control (25°C). Aliquots of 40 *μ*L were taken every 30 min for the analysis. All of the quantitative measurements were conducted using a Hitachi LaChrom Elite HPLC system (Hitachi High-Technologies, Tokyo, Japan) equipped with a quaternary pump (L-2130), an autosampler (L-2200), a column oven (L-2300), and a diode array detector (DAD/L-2450). Separation was performed using a TSKgel ODS-100Z reversed phase column (4.6 mm × 250 mm, 5 *μ*m, Tosho, Tokyo, Japan) with a mobile phase composed of acetonitrile-water-methanol-trifluoroacetic acid (72 : 18 : 10 : 0.5, v/v/v/v). The flow rate was 1.6 mL/min. The UV detector was set at 270 nm. Curve fitting was performed using Excel (MS Office 2013) to minimize the *R*^2^ value.

### 2.4. Cell Culture

HaCaT human keratinocyte cells [19] at passages 36 and 37 were purchased from Cell Lines Services (CLS, Heidelberg, Germany) and were grown in DME/F12 (0.07 mM Ca^2+^) supplemented with 2% FBS at 37°C in a humidified atmosphere of 5% CO_2_. The growth medium was prepared from DME/F12 (D9785; Sigma-Aldrich) by adding 0.07 mM Ca^2+^, 365 mg/L L-glutamine, 59.05 mg/L L-leucine, 91.25 mg/L L-lysine·HCl, 61.2 mg/L MgCl_2_·6H_2_O, 48.84 mg/L MgSO_4_ (anhydrous), 17.2 mg/L L-methionine, and 1.2 g/L NaHCO_3_ and was adjusted to pH 7.4 with 1 mM NaOH. For the experiments, the cells were seeded on a collagen-coated (Cellmatrix type IA) silicone stretch chamber (see the following) or 15 mm round glass coverslips (Matsunami, Osaka, Japan) and cultured in DME/F12 (1.05 mM Ca^2+^) supplemented with 10% FBS to allow cell attachment. After 1 day, the medium was replaced with DME/F12 (0.07 mM Ca^2+^) supplemented with 2% FBS, and the cells were further incubated for 1 day to achieve confluence.

The physiological experiments were performed as described previously [2]. A brief explanation follows.

### 2.5. Cell Stretch Experiments and Wound Closure Assay

The cells were cultured in a stretch chamber molded out of Silpot 184 W/C silicone elastomers (Dow Corning Toray, Tokyo, Japan). A chamber with cultured cells was attached to a stretching machine (NS-600W or ST-600W, STREX, Osaka, Japan) mounted on the stage of an inverted microscope (IX-70, Olympus, Tokyo, Japan) for intracellular Ca^2+^ imaging or an upright microscope (BX51WI, Olympus) for extracellular ATP imaging. HaCaT cells were seeded on collagen-coated silicone stretch chambers or 15 mm round glass coverslips at 3 × 10^5^ cells/cm^2^ and grown to confluence. A narrow cell-free gap (about 250 *μ*m) was created in a fully confluent monolayer by removing a silicone strip that was attached to the bottom of the stretch chamber during cell seeding. The wound closure process was monitored every 3 h after making the scratch using an inverted microscope (IX-70 Olympus) with a 4x (UPlanFL N, 0.13) objective. The wound closure speed was defined as the percentage of the wound closure area, which was calculated from the ratio of the final migrated area to the initial cell-free area.

### 2.6. Intracellular Ca^2+^ Measurement and Real-Time Imaging of the Released ATP

At 3 h after making a scratch, the HaCaT cells in the stretch chamber were loaded with 1 *μ*M Fluo-8 AM using 0.1–0.2% of Cremophor EL (Sigma-Aldrich) for 40–60 min in an incubator at 37°C. After washing away the dye with DME/F12 containing 2.0 mM Ca^2+^, the chamber with the cells was attached to the stretching device on the stage of an inverted microscope with 4x (UPlanFL N, 0.13) or 10x (UPlanFL 0.30) objectives. Time-lapse Fluo-8 fluorescence images were acquired at 0.5 s intervals using MetaMorph software (v6.3 and 7.5, Molecular Devices, Downingtown, PA).

The stretch-induced release of ATP was measured in real-time using the imaging system, as described previously [20]. Briefly, the luciferin-luciferase ATP bioluminescence was detected using a high-sensitivity camera system simultaneously with infrared DIC imaging to monitor exact cell locations and extension during stretching. At 3 h after making a scratch, the cells in the stretching chamber were attached to the stretching device on the stage of an upright microscope (BX51WI, Olympus) with a 4x objective (340 Fluor XL, 0.28) and the medium was replaced with DME/F12 medium (2.0 mM Ca^2+^ and 10 mM HEPES, pH 7.4) containing high-sensitivity luciferin-luciferase solution (60315; Lucifer HS Set, Kikkoman Biochemifa, Tokyo, Japan). Images were acquired using the MetaMorph software with a stream acquisition mode (exposure time 100 ms).

### 2.7. The Knockdown of TRPC6 by shRNA

TRPC6 shRNA plasmids that coexpressed RFP (TF308626; OriGene Technologies, Rockville, MD) were used. The shTRPC6 targeting sequence was 59-AAGCAGGACATCTCAAGTCTCCGCTATGA-39. A scrambled noneffective plasmid with the same nucleotide content was used as a negative control. Each shRNA at a concentration of 45 nM was transfected into HaCaT cells using Lipofectamine reagent, according to the manufacturer's instructions.

### 2.8. Ex Vivo Skin Preparation and Live Ca^2+^ Imaging of the Epidermis

Biopsies were taken from the outer forearm of a volunteer with atopic skin. Written informed consent was obtained from the volunteer. The study was approved by the Pixy Central Institute Ethics Committee, 2009. The sample of skin tissue was placed in PBS prior to treatment with dispase. After overnight digestion with 500 U/mL dispase in serum-free F12/DME with or without hyperforin/HP-*β*-CD at 4°C, the epidermis was peeled off from the dermis with forceps. The detached pieces of epidermis were fixed with intradermal needles on an elastic silicone chamber and were further incubated at 37°C in a humidified 5% CO_2_ atmosphere for 12 h. Epidermis tissue was loaded with 1 *μ*M Fluo-8 AM using 0.2% of Cremophor EL in culture media for 1 h at 37°C. After washing away the dye with DME/F12 containing 2.0 mM Ca^2+^, the chamber containing the cells was attached to a pulse-motor-driven stretching machine (NS-600W or ST-600W, STREX) mounted on the stage of an inverted laser scanning confocal microscope (LSM510 with a 10x lens, Carl Zeiss, Jena, Germany). Time-lapse Fluo-8 fluorescence and Nomarski differential interference contrast images were acquired at 1 s intervals. The Ca^2+^ imaging experiments were performed at room temperature (24 ± 2°C).

## 3. Results

### 3.1. Preparation of Stable Hydrophilic Hyperforin Encapsulated in HP-*β*-CD and Its Effect on Wound Closure

**Figure 1:**
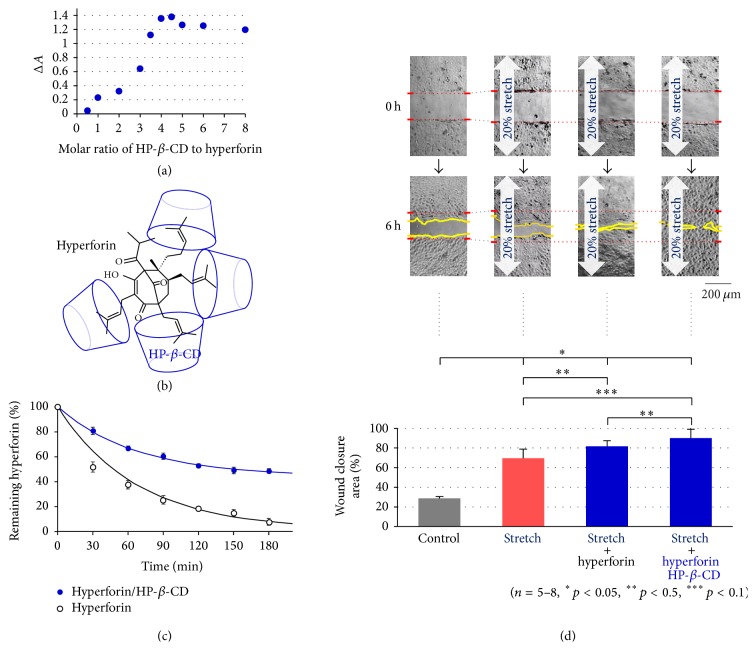
The complexation of hyperforin with hydroxypropyl-*β*-cyclodextrin (HP-*β*-CD) and its effects on wound closure in HaCaT cells. (a) The molar ratio graph obtained by UV/Vis spectra measurements of the inclusion complex formed by hyperforin (4.66 × 10^−4^ M) and HP-*β*-CD (0–8.0 equivalents) at 25°C. (b) A possible model of the 1 : 4 complex of hyperforin/HP-*β*-CD. (c) The photodegradation of hyperforin/HP-*β*-CD in aqueous solution and hyperforin in methanol induced by LED light exposure. Curve fitting was performed by single exponential decay with a baseline. The baselines obtained for hyperforin/HP-*β*-CD and hyperforin were 44% and 1.4%, respectively. (d) The effects of hyperforin and hyperforin/HP-*β*-CD treatments on wound closure in keratinocytes under sustained stretching. Stretch stimulation (20%) facilitated wound closure in HaCaT keratinocytes (stretch). Treatment with hyperforin (1 *μ*M) further accelerated the wound closure (stretch + hyperforin) and the wound gap was nearly closed at 6 h after scratching. Hyperforin/HP-*β*-CD (1 *μ*M as hyperforin) showed equal or greater efficacy to hyperforin (stretch + hyperforin/HP-*β*-CD) in promoting wound closure. The data are shown as representative DIC images (upper pictures at 0 h and 6 h) and by the averages of the calculated percentage of the wound closure areas (lower graph). The quantitative data in (c) and (d) are shown as the mean ± SEM.

To improve the photostability and aqueous solubility of hyperforin, it was molecularly encapsulated in cyclodextrin. Hyperforin was complexed with hydroxypropyl-*β*-cyclodextrin (HP-*β*-CD) at different molar ratios using the solvent evaporation method. The hyperforin/HP-*β*-CD complexes were investigated with UV/Vis spectroscopy in aqueous solution. The molar ratio method was used to determine the stoichiometry of the inclusion complex formed by hyperforin and HP-*β*-CD. Δ*A*, the difference in the absorbance of hyperforin with and without HP-*β*-CD, was plotted against the molar ratio of HP-*β*-CD to hyperforin at 280 nm (Figure 1(a)). The curve for hyperforin/HP-*β*-CD showed an inflexion point at a ratio of 1 : 4, suggesting that the inclusion complex formed HP-*β*-CD tetracapped hyperforin at hemiterpene terminal moieties (Figure 1(b)). Next, we checked the light stability of a 1 : 4 complex of hyperforin/HP-*β*-CD using HPLC. Figure 1(c) shows the visible light-induced degradation curves of hyperforin and the hyperforin/HP-*β*-CD complex. The apparent half-life of hyperforin was 30 min, while that of the hyperforin/HP-*β*-CD complex was prolonged to 180 min. A curve fitting analysis by single exponential decay with a baseline showed a large baseline (44%) in the curve for hyperforin/HP-*β*-CD, suggesting the existence of a nondegraded (photoprotected) form in the complex (Figure 1(c) fitting line).

We previously demonstrated that the wound closure of keratinocytes was accelerated by stretching and that hyperforin treatment further enhanced the effect [2] (Figure 1(d)). In the present study, we examined the effect of hyperforin/HP-*β*-CD on wound closure. A confluent monolayer of HaCaT cells cultured on silicone membrane was linearly scratched to create a cell-free gap of ~250 *μ*m width, and the wound was allowed to heal under various conditions. Figure 1(d) shows representative wound closing images captured at 0 and 6 h after scratching and the average of the calculated percentage of the wound closure area. A 20% sustained stretch facilitated wound closure in comparison to nonstretched cells (control) and hyperforin (1 *μ*M) treatment further enhanced the effect of stretching, as shown previously. Hyperforin/HP-*β*-CD (1 *μ*M) was equally (or more) effective in facilitating wound closure.

### 3.2. The Stretch-Induced ATP Release and the Initiation of Ca^2+^ Waves from the Leading Cells on the Wound Gap

**Figure 2:**
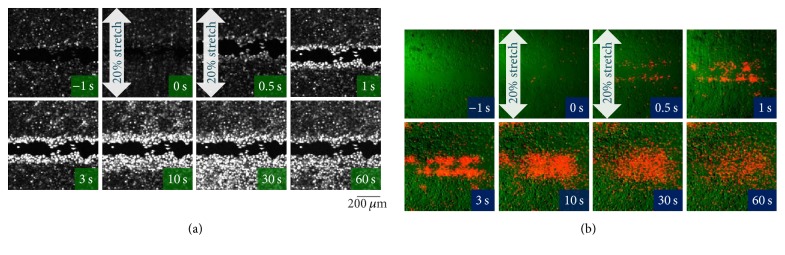
Stretch-induced Ca^2+^ wave propagation from the wound edge and ATP release from the leading cells. Hyperforin/HP-*β*-CD-treated HaCaT cells at 3 h after scratching were subjected to a single stretch (20% for 1 s), which was applied perpendicular to the linear gap. (a) The intracellular Ca^2+^ responses were measured using the Ca^2+^ fluorescence indicator, Fluo-8. In response to the stretch, the cells at the leading edge exhibited a remarkably long-lasting increase in intracellular Ca^2+^, and the Ca^2+^ increase subsequently propagated to the cells located behind the edge (Movie S1 online). (b) The release of ATP was visualized using a real-time luciferin-luciferase bioluminescence imaging system. Representative overlay images of the ATP-dependent luminescence (red) and infrared DIC images (green) are shown. Following stretching, the release of ATP was only observed in the cells at the leading edge. The released ATP diffused into the entire area and remained at a high concentration for several minutes (Movie S2 online).

It was reported that stretch stimulation induced intercellular Ca^2+^ waves in hyperforin-treated HaCaT cells, which were initiated from the leading cells on the wound edge and that this occurred due to the release of ATP from the leading cells and the activation of TRPC6 on the cells behind the leading edge through the activation of P2Y with the spread ATP [2]. We assessed whether hyperforin/HP-*β*-CD also has the same effects on HaCaT keratinocytes. At 3 h after making a narrow scar on the confluent monolayer of hyperforin/HP-*β*-CD-treated cells, stretching (20% for 1 s, perpendicular to the linear gap) induced an increase in the intracellular Ca^2+^ in almost all of the leading cells on the wound edge and the Ca^2+^ increase propagated towards the rear cells behind the edge in a wave-like pattern (Figure 2(a); Movie S1) (see Supplementary Material available online at https://doi.org/10.1155/2017/8701801). The Ca^2+^ waves occurred due to the release of ATP from the leading cells and its diffusion to the surrounding cells behind the edge, as shown in Figure 2(b) (Movie S2). The stretch applied parallel to the linear gap had essentially the same effect on ATP-Ca^2+^ signaling and wound healing in HaCaT cells [2].

One notable advantage of hyperforin/HP-*β*-CD was that the effects on the stretch-induced ATP and Ca^2+^ signaling were more reproducible than those obtained simple hyperforin. This may be attributed to the improvement of photostability and the aqueous solubility of hyperforin/HP-*β*-CD.

### 3.3. The Pharmacological Analysis of the Stretch-Induced Ca^2+^ Responses and Wound Closure in Hyperforin/HP-*β*-CD-Treated Cells

**Figure 3:**
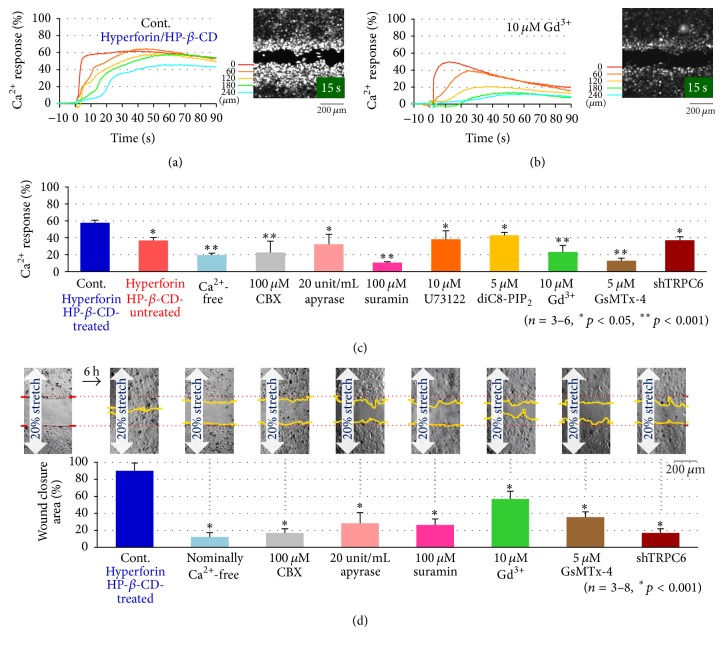
The effects of various inhibitors on the stretch-induced Ca^2+^ responses and wound closure. (a) The time course of changes in the fluorescence intensity of Fluo-8 due to a transient 20% stretch in hyperforin/HP-*β*-CD-treated HaCaT cells (control). Each color trace indicated the data at different distances of 0, 60, 120, 180, and 240 from the wound edge (inset image). The intensity was normalized to the peak value obtained with ionomycin treatment at the end of each experiment. (b) The effects of Gd^3+^ on the stretch-induced Ca^2+^ response as a typical example of the blocking effects of the inhibitors. Gd^3+^ (10 *μ*M) was applied at 10 min before the application of a 20% stretch. (c) The effects of various inhibitors on 20% transient stretch-induced Ca^2+^ responses in hyperforin/HP-*β*-CD-treated HaCaT cells. The intensity traces at each distance from the wound edge were averaged and normalized to the peak intensity obtained with ionomycin treatment. The data show the average of the peak values obtained in 3–6 separate experiments. Various inhibitors, including CBX (100 *μ*M), apyrase (20 Unit/mL), suramin (100 *μ*M), U73122 (10 *μ*M), diC8-PIP_2_ (5 *μ*M), Gd^3+^ (10 *μ*M), and GsMTx-4 (5 *μ*M), were applied at 10 min before the stretch stimulation. A Ca^2+^-free condition was achieved by changing the medium to Ca^2+^-free medium that contained 0.5 *μ*M EGTA. All of the quantitative data are shown as the mean (±SEM). (d) The effects of various inhibitors on the stretch facilitated wound closure in hyperforin/HP-*β*-CD-treated HaCaT cells. Confluent cell cultures were scratched and allowed to migrate for 6 h under a sustained 20% stretch in a medium that contained various inhibitors, including CBX (100 *μ*M), apyrase (20 Unit/mL), suramin (100 *μ*M), Gd^3+^ (10 *μ*M), and GsMTx-4 (5 *μ*M) or in nominally Ca^2+^-free medium. shTRPC6 was applied to the cells for 3 h; the cells were then grown to confluence. Representative DIC images (upper panel) and the means of 3–8 wound closure experiments at 6 h after scratching (lower panel) are shown. All of the quantitative data are shown as the mean (±SEM).

To analyze the characteristics of the stretch-induced Ca^2+^response, the effects of various inhibitors on the Ca^2+^response were evaluated in hyperforin/HP-*β*-CD-treated HaCaT cells. The time course of the intracellular Ca^2+^response induced by a 20% stretch was measured at different distances (0–240 *μ*m) from the scar (Figure 3(a), control; hyperforin/HP-*β*-CD-treated cells). At 0 *μ*m (wound edge), the Ca^2+^response was evoked immediately after stretching and it was prolonged by several min. When the distance from the edge was increased, a longer time lag was found before the onset of the activity; however, the amplitudes of the plateau phase were nearly the same. These results were consistent with the idea that Ca^2+^ waves caused by the simple diffusion of ATP released from the leading cells at wound edge and the activation of P2Y in the surrounding cells behind the wound edge. When Gd^3+^ (10 *μ*M), an inhibitor of the stretch-activated channel, was applied, the Ca^2+^ response in the peak was reduced and the rate of decay was obviously faster, especially at the distant regions (Figure 3(b)). The Ca^2+^ responses were similarly measured under various conditions and inhibitors and were evaluated by the peak response in an averaged trace of the responses at different distances (Figure 3(c)). The suppression observed in Ca^2+^-free medium, in hyperforin/HP-*β*-CD-untreated cells and in shTRPC6-treated cells, suggested the involvement of the influx of Ca^2+^via TRPC6. The inhibition by the treatments with suramin (P2-receptor antagonist, 100 *μ*M), apyrase (ATP-hydrolyzing enzyme, 20 U/mL), and CBX (hemichannel blocker, 100 *μ*M) suggested the contribution of ATP signaling in this process. The reduction by each treatment with U73122 (PLC inhibitor, 10 *μ*M) and diC8-PIP2 (a water-soluble PIP_2_ analog that suppresses the activity of PLC by competing with PIP_2_, 10 *μ*M) suggested that the P2Y receptor-Gq-PLC-DAG-mediated signaling cascade was involved in the activation of TRPC6. These results were the same as those obtained by treatment with hyperforin (nonencapsulate) and stretch stimulation [2]. This suggests the involvement of the release of ATP via hemichannels in the leading cells and that the activation of P2Y in the cells behind the wound edge prolonged the influx of Ca^2+^ via TRPC6 through the Gq-PLC-DAG cascade.

To confirm whether hyperforin/HP-*β*-CD facilitates wound closure by amplifying ATP-Ca^2+^ signaling, we assessed the effects of the various inhibitors that were used above on the wound closure during sustained stretching. The wound gap was almost closed at approximately 6 h (Figure 3(d), control) after scratching, while treatment with Ca^2+^ depletion (nominally Ca^2+^-free), CBX (100 *μ*M), apyrase (20 U/mL), suramin (100 *μ*M), Gd^3+^(10 *μ*M), GsMTx-4 (5 *μ*M), and shTRPC6 treatment delayed wound closure (Figure 3(d)). This suggested that the wound closure process required ATP-Ca^2+^ signaling, especially the influx of Ca^2+^ through TRPC6.

### 3.4. The Effects of Hyperforin/HP-*β*-CD Treatment on the Ca^2+^ Responses in the Ex Vivo Skin of Atopic Dermatitis

**Figure 4:**
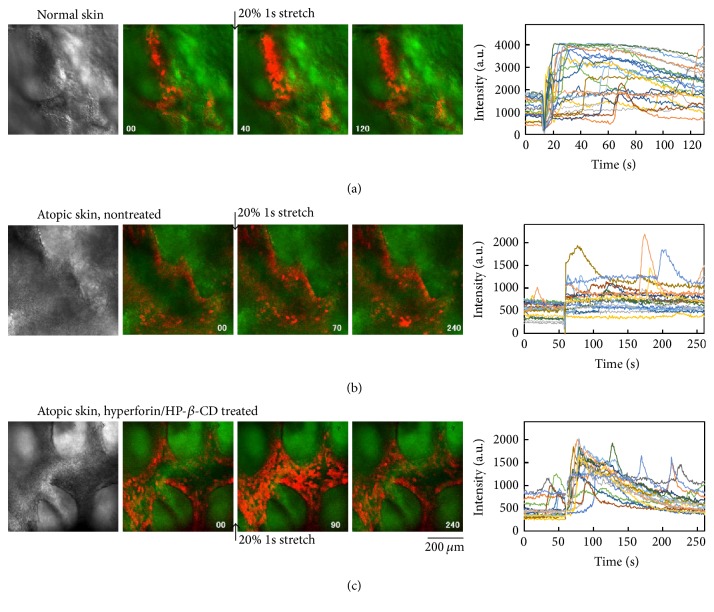
The effects of hyperforin/HP-*β*-CD treatment on the Ca^2+^dynamics in the epidermis of atopic skin ex vivo. The epidermis was detached from the dermis by dispase treatment and fixed on a stretch chamber with intradermal needles. The fluorescence of the Ca^2+^ indicator, Fluo-8AM, was observed with a laser confocal microscope (left image panels) and the changes in intensity in several cells were plotted in the right panels. (a) The Ca^2+^ oscillation and the Ca^2+^ response to stretch stimulation (20%, 1 s, transient) in the epidermis of normal skin ex vivo. Frequent Ca^2+^ oscillation and a large Ca^2+^ response to stretching and subsequent Ca^2+^ waves were prominent (see also Movie S3). (b) In the atopic epidermis, little Ca^2+^ oscillation and a very weak Ca^2+^ response to stretching were observed (see also Movie S4). (c) The 24 h treatment of atopic skin with hyperforin/HP-*β*-CD drastically induced autonomous Ca^2+^ oscillation and led to a transient, long-lasting Ca^2+^ increase induced by stretching (see also Movie S5).

Next, we assessed the effects of hyperforin/HP-*β*-CD treatment and stretch mechanical stimulation on an ex vivo epidermis of atopic dermatitis. The epidermis, which was detached from the dermis after overnight treatment with dispase, was loaded with Fluo-8AM and observed with a laser confocal microscope. Normal skin exhibited frequent spontaneous Ca^2+^ oscillations and a large Ca^2+^ response to stretch stimulation (1 s single) and subsequent Ca^2+^ waves with the long-lasting elevation of Ca^2+^ (Figure 4(a), Movie S3). In contrast, the epidermis of atopic dermatitis showed few oscillations and only a small response to stretching without any waves (Figure 4(b), Movie S4). In contrast, atopic skin that had been treated with hyperforin/HP-*β*-CD for 24 h exhibited autonomous Ca^2+^ oscillation, and a transient long-lasting increase in Ca^2+^ and more frequent Ca^2+^oscillations following stretch stimulation (Figure 4(c), Movie S5). The application of hyperforin/HP-*β*-CD-treatment to atopic skin for 24 h led to the recovery of the mechanosensitive ATP-Ca^2+^ signaling, which was dysfunctional in the untreated atopic epidermis.

## 4. Discussion

The topical application of hyperforin, which is a traditional folk remedy, has anti-inflammatory, antioxidative, antibacterial, antinociceptive, and wound healing effects. Recently, accumulating evidence indicates that hyperforin facilitates the keratinocyte differentiation caused by the uptake of Ca^2+^ through TRPC6 [11]. Our previous studies showed that hyperforin-treated HaCaT keratinocytes could accelerate wound closure in conjunction with exogenous and endogenous mechanical stretching through the facilitation of the ATP-Ca^2+^signaling cascade [2]. The impact of hyperforin on mechanosensitivity remains unclear, but hyperforin certainly amplifies ATP-Ca^2+^signaling and facilitates reepithelialization during wound healing. However, due to the photoinstability of hyperforin, daylight initiates its facile oxidative degradation [21]. Prenyl side chains (hemiterpene moieties) containing conjugated double bonds are generally prone to oxidation. In order to enhance the stability of hyperforin and exert its topical therapeutic potential, hyperforin was encapsulated by forming a supramolecular complexation with HP-*β*-CD. The solubility of HP-*β*-CD is highest in ethanol as well as water among the CD compounds, *α*-CD, *β*-CD, methylated-*β*-CD, sulfobutyl ethyl-*β*-CD, *γ*-CD, and HP-*β*-CD. This amphipathic property was a major advantage when making the inclusion complex with hydrophobic hyperforin. The molar ratio method indicated that the optimal ratio of the hyperforin/HP-*β*-CD complex was 1 : 4. This meant the formation of HP-*β*-CD-tetracapped hyperforin, where the hyperforin was encapped with HP-*β*-CD at each hemiterpene moiety [22] as shown in Figure 1(b). The novel inclusion complex showed obvious photostability in comparison to hyperforin (Figure 1(c)). The curve fitting of the decay time course of hyperforin/HP-*β*-CD indicated the existence of a large nondecayed component that corresponded to photostable hyperforin. This modification can contribute to both pharmaceutical application and topical medication.

We assessed the effects of hyperforin/HP-*β*-CD on wound healing and ATP-Ca^2+^ signaling in keratinocytes. Hyperforin/HP-*β*-CD enhanced the acceleration of wound closure by stretching with a similar efficiency to hyperforin (Figure 1(d)). In hyperforin/HP-*β*-CD-treated keratinocytes, stretching induced a conspicuous increase in the Ca^2+^ levels in the leading cells facing the wound edge and the Ca^2+^ waves slowly propagated to the cells behind the wound edge (Figure 2(a)). These propagating Ca^2+^ waves were entirely due to the release of ATP from the leading cells (Figure 2(b)). The pathway of ATP release was CBX sensitive (Figure 3(c)) and presumably pannexin hemichannels from our previous study [2]. The migrating cells at the wound edge represented morphological changes that were similar to those observed at the epithelial-to-mesenchymal transition and might be more susceptible to endogenous and exogenous mechanical stress [2, 23]. The increase in Ca^2+^ in the cells behind the wound edge was dependent on the influx of Ca^2+^ via the TRPC6 channels, which were activated by the activation of P2Y through the Gq-PLC-DAG-mediated signaling cascade (Figure 3(c)) [2] and lasted for a relatively long period. The concentration and duration of the Ca^2+^ increase were dependent on the distance from the wound edge, making a Ca^2+^ gradient from the leading cells to the following cells. This Ca^2+^ gradient may be essential for organized wound healing, including cell migration, molecular relocation, and gene expression. The cell traction of the cells located behind the edge by migrating leading cells is also an important mechanical cue for wound healing that is controlled by Ca^2+^ dependent cell-cell interaction molecules such as E-cadherin. This Ca^2+^ signaling is enhanced by treatment with hyperforin/HP-*β*-CD.

Interestingly, the reagents that blocked the increase in Ca^2+^ also suppressed the acceleration of wound closure in response to stretching in hyperforin/HP-*β*-CD-treated cells (Figure 3(d)). Thus, the hyperforin/HP-*β*-CD complex shows a similar efficiency to hyperforin in inducing mechanosensitive ATP-Ca^2+^ signaling and wound closure in keratinocytes. In fact, hyperforin/HP-*β*-CD seems to be superior due to the reproducibility of the data concerning stretch-induced ATP and Ca^2+^ signaling, which may be attributed to the photostability and aqueous solubility of hyperforin/HP-*β*-CD.

In our experimental design, HaCaT keratinocytes were cultured under low extracellular Ca^2+^ conditions (0.07 mM), which mimicked the extracellular Ca^2+^ environment for barrier-perturbed epidermis, such as the environment that would result from skin stripping or the use of surfactants. Atopic dermatitis is also a skin barrier dysfunction. Topical medication of hyperforin-rich St. John's wort cream has been shown to be effective in patients with atopic dermatitis [10–14]. The analysis of the laser scanning microscopy images has shown that the hyperforin-rich cream reduces the skin surface dryness and improves the moisture level of the stratum corneum [14]. However, the mechanism underlying the improvement of symptoms in atopic skin remains unclear. Our present study is the first to demonstrate how the Ca^2+^dynamics of atopic skin behave under mechanical environments such as wound healing and reepithelialization. We observed the Ca^2+^ dynamics induced by the stretching of epidermis ex vivo using a confocal microscope (Figure 4). In atopic epidermis, there was a remarkable decrease in the Ca^2+^ responses and oscillations induced by stretching (Figure 4(b)). Treatment with hyperforin/HP-*β*-CD for 24 h restored the Ca^2+^ responses and oscillations, even in atopic skin (Figure 4(c)). These results suggest that the pathogenesis of atopic dermatitis is related to ATP-Ca^2+^ signaling and that hyperforin/HP-*β*-CD may have therapeutic application in the treatment of atopic dermatitis.

## 5. Conclusions

Cutaneous wound healing is accelerated by mechanical stress both exogenously and endogenously, and treatment with hyperforin enhances the acceleration through the facilitation of ATP-Ca^2+^ signaling in keratinocytes. We succeeded in making HP-*β*-CD-tetracapped hyperforin (hyperforin/HP-*β*-CD), which possessed increased aqueous solubility and improved photoprotection. Treatment with hyperforin/HP-*β*-CD enhanced the mechanically induced ATP-Ca^2+^ signaling and accelerated wound closure in HaCaT keratinocytes with equal (or greater) efficacy to hyperforin. We also applied hyperforin/HP-*β*-CD on atopic skin ex vivo and found that hyperforin/HP-*β*-CD treatment for 24 h improved the stretch-induced Ca^2+^ responses and oscillations, which reduced in atopic skin. The data suggest that hyperforin/HP-*β*-CD is a potent targeted therapeutic agent that can be used to promote epidermal wound healing and to treat atopic dermatitis.

## Supplementary Material


Supplemental Movies
S1. The stretch-induced Ca^2+^ responses and the propagation of Ca^2+^ waves from the wound edge.
S2. The stretch-induced release of ATP from the leading cells of the wound edge (20% for 1 s, applied perpendicular to the linear gap).
S3. The Ca^2+^ oscillations and the Ca^2+^ response to stretching in the epidermis of normal skin *ex vivo. *
S4. The reduced Ca^2+^ response to stretching in the epidermis of atopic skin *ex vivo*.
S5. The hyperforin/HP-β-CD treatment-enhanced Ca^2+^ oscillations and Ca^2+^ response to stretching in the epidermis of atopic skin *ex vivo*.
